# Hashimoto's thyroiditis – an independent risk factor for papillary carcinoma^☆^

**DOI:** 10.1016/j.bjorl.2017.08.012

**Published:** 2017-09-14

**Authors:** Barbora Uhliarova, Andrej Hajtman

**Affiliations:** a FD Roosevelt Faculty Hospital, Department of Otorhinolaryngology, Banska Bystrica, Slovakia; b Comenius University, Jessenius Faculty of Medicine, Department of Otorhinolaryngology, Head and Neck Surgery, Martin, Slovakia

**Keywords:** Thyroid cancer, Microcarcinoma, Hashimoto's thyroiditis, Câncer de tireoide, Microcarcinoma, Tireoidite de Hashimoto

## Abstract

**Introduction:**

The link between Hashimoto's thyroiditis and thyroid carcinoma has long been a topic of controversy.

**Objective:**

The aim of our study was to determine the prevalence of thyroid carcinoma and Hashimoto's thyroiditis coexistence in histopathologic material of thyroidectomized patients.

**Methods:**

In a retrospective study, the clinicohistopathologic data of 2117 patients (1738 females/379 males), who underwent total or partial thyroidectomy for thyroid gland disorder at a single institution from the 1st of January 2005 to the 31st of December 2014 were analyzed.

**Results:**

Thyroid carcinoma was detected in 318 cases (15%) and microcarcinoma (thyroid cancer ≤10 mm in diameter) was found in permanent sections in 169 cases (8%). Hashimoto's thyroiditis was detected in 318 (15%) patients. Hashimoto's thyroiditis was significantly more often associated with thyroid carcinoma and microcarcinoma compare to benign condition (*p* = 0.048, *p* = 0.00014, respectively). Coexistence of Hashimoto's thyroiditis and thyroid carcinoma/thyroid microcarcinoma did not affect tumor size (*p* = 0.251, *p* = 0.098, respectively), or tumor multifocality (*p* = 0.831, *p* = 0.957, respectively). Bilateral thyroid microcarcinoma was significantly more often detected when Hashimoto's thyroiditis was also diagnosed (*p* = 0.041), but presence of Hashimoto's thyroiditis did not affect bilateral occurrence of thyroid carcinoma (*p* = 0.731).

**Conclusion:**

Hashimoto's thyroiditis is associated with significantly increased risk of developing thyroid carcinoma, especially thyroid microcarcinoma.

## Resumo

**Introdução:**

A relação entre a tireoidite de Hashimoto e o carcinoma de tireoide tem sido um tema de controvérsia por um longo tempo.

**Objetivo:**

Determinar a prevalência da coexistência de carcinoma de tireoide e tireoidite de Hashimoto no exame histopatológico de amostras de pacientes tireoidectomizados.

**Método:**

Em um estudo retrospectivo, foram analisados os dados clinico-histopatológicos de 2.117 pacientes (1.738 mulheres/379 homens), submetidos à tireoidectomia total ou parcial por distúrbio da glândula tireoide em uma única instituição, de 1° de janeiro de 2005 a 31 de dezembro de 2014.

**Resultados:**

O carcinoma de tireoide foi detectado em 318 casos (15%) e o microcarcinoma (câncer de tireoide ≤ 10 mm de diâmetro) foi encontrado em secções permanentes em 169 casos (8%). A tireoidite de Hashimoto foi detectada em 318 (15%) pacientes e foi associada ao carcinoma da tireoide e ao microcarcinoma com maior frequência em comparação com condições benignas (*p* = 0,048, *p* = 0,00014, respectivamente). A coexistência de tireoidite de Hashimoto e carcinoma/microcarcinoma não influenciou o tamanho do tumor (*p* = 0,251, *p* = 0,098, respectivamente) ou a multifocalidade tumoral (*p* = 0,831, *p* = 0,957, respectivamente). O microcarcinoma de tireoide bilateral foi detectado com maior frequência quando a tireoidite de Hashimoto também foi diagnosticada (*p* = 0,041), mas a presença de tireoidite não influenciou na ocorrência bilateral de carcinoma (*p* = 0,731).

**Conclusão:**

A tireoidite de Hashimoto está associada a um aumento significativo do risco do desenvolvimento de carcinoma de tireoide, especialmente microcarcinoma da tireoide.

## Introduction

Thyroid cancer is the most common malignancy of endocrine system and accounts for approximately 1% of all cancers. The worldwide incidence of thyroid carcinoma (TC) has increased, however, the cause of this increase is not clear. It is questionable, whether this increase is absolute or the result of improved diagnostic capabilities.^1^, ^2^ Moreover, the incidental finding of thyroid microcarcinoma (TMC) (thyroid cancer ≤10 mm in diameter) during pathological examination of the resection specimens in patients operated for benign thyroid condition remains a common scenario, in spite of advances in preoperative investigation, mainly Ultrasound (US) and Fine Needle Aspiration Cytology (FNAC).^3^, ^4^, ^5^

TMCs are found at rates of 0.5–35.6% at autopsy or in surgical specimens where carcinoma had been unsuspected.^6^, ^7^

Hashimoto's thyroiditis (HT) is a chronic lymphocytic thyroiditis, and the most common cause of hypothyroidism in areas with proper amounts of iodine.^8^ HT was first described in 1912 by Hakaru Hashimoto,^9^ a Japanese surgeon and pathologist, as an autoimmune disease, affecting 5% of the general population. The pathology is characterized by diffuse lymphocyte infiltration, fibrosis, and parenchymal atrophy. Although the link between chronic inflammation and cancer is well established, the association between HT and Papillary Thyroid Carcinoma (PTC) has been controversial in literature since its initial description by Dailey et al. in 1955.^10^ The aim of our study was to determine the prevalence of TC and HT coexistence in histopathologic material of thyroidectomized patients.

## Methods

The retrospective study was conducted with patients surgically treated at a single institution from the 1st of January 2005 to the 31st of December 2014 for thyroid gland disorder. The study was approved by the Ethics Committees. All patients signed their informed consents.

Thyroid ultrasound was performed in all patients. The extension of the thyroidectomy was decided by the endocrinologist and operating surgeon, depending on the extension of lesions, patient's approval and intraoperative findings.

Type of thyroid gland disease was determined by histopathological examination. We studied the following parameters: histopathologic findings, presence of Hashimoto's thyroiditis, size of the tumor, multifocality, bilateral presentation.

Pathological classification was performed for all thyroid specimens according to World Health Organization guidelines.^11^ The histological criteria used to make a diagnosis of HT included diffuse lymphoplasmacytic infiltration, germinal centers, and enlarged epithelial cells with large nuclei and eosinophilic cytoplasm (Askanazy or Hürthle cells). Non-specific lymphocytic thyroiditis occurring immediately adjacent to a tumor could not be differentiated from perineoplastic inflammation and was not included in number with HT to avoid over-diagnosis in our study. Hashimoto's thyroiditis was classified in one single grade. To set up the diagnosis of HT, pathologists from one single center were involved. Pathological specimens were collected from medical charts.

The largest tumor size was recorded. Patients were categorized as TMC if the largest tumor diameter was ≤1 cm. Multifocal thyroid cancer was defined as two or more tumor sites (within one or both lobes of thyroid gland). Multifocal occurrence of thyroid tumor in both lobes of thyroid gland was classified as bilateral.

The accuracy of fine needle aspiration cytology (FNAC) in the differential diagnosis of thyroid nodules was also. In patients with multiple nodules, one dominant nodule or nodule with features for malignancy on USG (hypoechogenicity, microcalcifications, absence of peripheral halo, irregular borders, intranodular hypervascularity and regional lymphadenopathy) was investigated. Thyroid FNAC result was classified using the Bethesda system for reporting thyroid cytopathology^12^: (I) Non-Diagnostic/Unsatisfactory (ND/UNS); (II) Benign; (III) Atypia of Undetermined Significance/Follicular Lesion of Undetermined Significance (AUS/FLUS); (IV) Follicular Neoplasm/Suspicious for Follicular Neoplasm (FN/SFN); (V) Suspicious For Malignancy (except of follicular carcinoma) (SFM); (VI) Malignancy.

The statistical analysis was performed with STATISTICA Cz 10. Frequencies of categorical data were tabulated and evaluated with Chi-square test using Yates's correction. For ordinal data, median and interquartile ranges were calculated and tested with the Kruskal–Wallis test, Mann–Whitney test or two-factorial ANOVA with post hoc Duncan test. A *p* < 0.05 was regarded as statistically significant.

## Results

A total of 2117 patients underwent partial or total thyroidectomy for thyroid disorder. There were 1738 (82%) female (mean age 46.5 ± 19.7 years) and 379 (18%) male (mean age 46.4 ± 20.9 years) in the study group.

Thyroid carcinoma was detected in 318 cases (15%) and microcarcinoma was found in permanent sections in 169 cases (8%). There were no differences in the gender (*p* = 0.122) of patients between groups (benign condition vs. TC vs. TMC). Patients with malignant tumor were significantly younger compare to patients with benign disorder of thyroid gland (*p* = 0.002) and PTMC (*p* = 0.003) (Table 1).

**Table 1:** General characteristics of study population.

	Benign	Thyroid carcinoma	Microcarcinoma	*p*-value
*N*	1630 (77%)	318 (15%)	169 (8%)	NA
Male	277 (17%)	60 (19%)	42 (24%)	0.122
Female	1348 (83%)	261 (81%)	129 (76%)	0.122
Age	47.2 ± 19.9	36.1 ± 14.7	54.5 ± 17.8	0.025
Multifocality	NA	38 (12%)	49 (29%)	0.015
Bilaterality	NA	32 (10%)	34 (20%)	0.047
PTC	NA	197 (62%)	159 (94%)	0.005
FVPTC	NA	74 (23%)	7 (4%)	0.044
FTC	NA	25 (8%)	3 (2%)	0.034
Others	NA	22 (7%)	0	0.008
HT+	163 (10%)	57 (18%)	98 (58%)	0.001

*N*, number of patient; NA, not applicable; PTC, papillary thyroid carcinoma; FVPTC, follicular variant of papillary thyroid carcinoma; FTC, follicular thyroid carcinoma; HT+, Hashimoto's thyroiditis present. Data are shown as median ± SD.

Papillary carcinoma was the most common histologic type in TC (62%) and TMC group (94%). Other histopathologic types of thyroid malignant tumors were significantly more often detected in TC compare to TMC group (Table 1).

**Figure 1:**
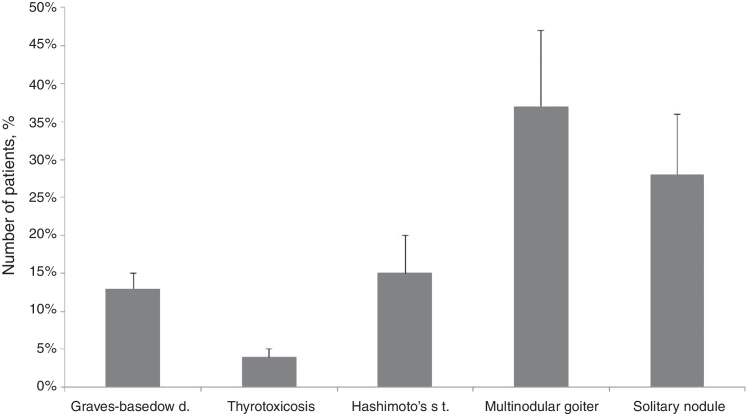
Thyroid disorders – indications for surgery. Data are shown as median ± SD.

Multinodular goiter (37%) and solitary nodule of thyroid gland (28%) were the most frequent indications for surgery (Fig. 1). Hashimoto's thyroiditis was detected in 318 (15%) patients. HT was significantly more often associated with thyroid carcinoma and microcarcinoma compare to benign condition (*p* = 0.048, *p* = 0.00014, respectively).

**Figure 2:**
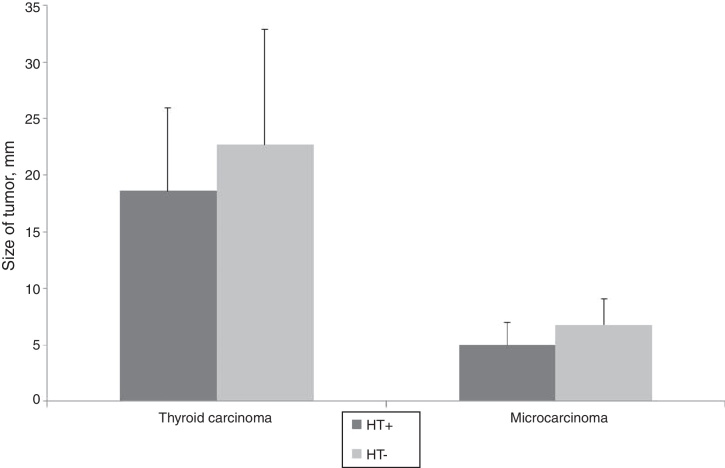
Tumor size according to the presence of Hashimoto's thyroiditis (HT+, Hashimoto's thyroiditis present; HT−, Hashimoto's thyroiditis absent; data are shown as median ± SD).

The mean tumor diameter was 5.8 ± 2.9 mm in TMC group and 19.6 ± 10.5 in TC group. There were no significant differences in tumor size according to the presence of HT in both groups (*p* = 0.098, *p* = 0.251, respectively) (Fig. 2).

**Figure 3:**
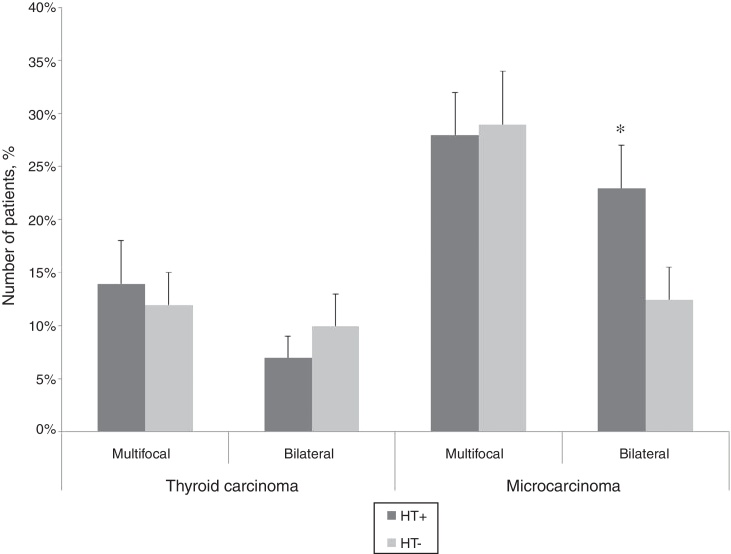
Prevalence of multifocality and bilaterality according to the presence of Hashimoto's thyroiditis (HT+, Hashimoto's thyroiditis present; HT−, Hashimoto's thyroiditis absent; data are shown as median ± SD, *p < 0.05).

Multifocal identification of malignancy was not associated to the accompanying Hashimoto's thyroiditis (TC: *p* = 0.831, TMC: *p* = 0.957). Bilateral detection of TMC was significantly more often detected when HT was also diagnosed (*p* = 0.041), but presence of HT did not affect bilateral occurrence of TC (*p* = 0.731) (Fig. 3).

Fine needle aspiration cytology was performed in 55% of patients with nodular goiter. The most frequent indication for FNAC was solitary thyroid nodule (50%). FNAC was indicated in 44% of patients with HT and in 32% without HT (*p* = 0.371). Results of FNAC according to the presence of HT are presented in Table 2. High sensitivity and specifity in the diagnosis of malignant thyroid nodule was observed by using aspiration cytology (HT present: 67%, 92%, respectively; HT absent: 82%, 97%, respectively). The accuracy of FNAC in the diagnosis of malignancy was significantly higher in patients without HT (93%) compare to patients with HT (82%) (*p* = 0.031).

**Table 2:** Results of fine needle aspiration cytology of thyroid nodules.

	HT+			HT−		
	FNAC	Definitive histology		FNAC	Definitive histology	
		Benign	Malignant		Benign	Malignant
ND/UNS	6%	0%	13%	6%	7%	5%
Benign	43%	60%	20%	48%	59%	12%
AUS/FLUS	20%	25%	13%	18%	18%	15%
FN/SFN	11%	10%	13%	13%	14%	10%
SFM	11%	5%	20%	7%	2%	25%
Malignant	9%	0%	21%	8%	0%	33%

FNAC, fine needle aspiration cytology; ND/UNS, nondiagnostic/unsatisfactory; AUS/FLUS, atypia of undetermined significance/follicular lesion of undetermined significance; FN/SFN, follicular neoplasm/suspected for a follicular neoplasm; SFM, suspected for malignancy; HT+, Hashimoto's thyroiditis present; HT−, Hashimoto's thyroiditis absent.

All patients in the study group were treated surgically by hemithyroidectomy or total thyroidectomy. When hemithyroidectomy was initial treatment and pathology revealed malignant tumor (TC or TMC), all patients underwent completion thyroidectomy 6–32 days (median 19 ± 8 days) after initial surgery. The final pathology from the remnant thyroid lobe after completion thyroidectomy detected bilateral malignant tumor in 14% (TC 4%, TMC 33%).

## Discussion

Hashimoto's thyroiditis, also called chronic lymphocytic or autoimmune thyroiditis, is part of the spectrum of autoimmune thyroid diseases and is associated with various degrees of thyroid hypofunction and circulating antibodies to thyroid antigens. The cause of HT is thought to be a combination of genetic susceptibility and environmental factors.^13^

Several studies showed the association of Hashimoto's thyroiditis and thyroid carcinoma.^1^, ^8^, ^10^, ^14^, ^15^, ^16^ A meta-analysis showed that the HT incidence is 2.77 higher in patients with PTC when compared to patients with benign thyroid diseases. In addition, in patients with thyroid carcinoma, the association of HT is 1.99 times higher among those with PTC than in patients with other pathological types of thyroid cancer.^17^ Indirectly, these findings may suggest a predisposition of HT patients to the development of PTC. In the present study, 18% of the cases showed both HT and TC, and even up to 58% cases of TMC were associated with HT. The mechanism by which Hashimoto's thyroiditis is associated with thyroid malignancy is not fully understood. One of the possible explanations is based on DNA damage caused by chronic inflammation. The inflammatory response may cause DNA damage through formation of reactive oxygen species, resulting in mutations that eventually lead to the development of PTC.^18^ The relationship between thyroid autoimmunity and cancer remains controversial. In a retrospective non-randomized study,^19^ it was shown that the presence of thyroglobulin antibody is an independent factor in the development of PTC.

RET/PTC, which is a RET rearrangement, a gene which codes a tyrosine-kinase receptor, is described as one of the mechanisms responsible for the association of HT and PTC.^20^ This oncogene was found in the large majority of tissues with HT and without detectable PTC, which may show a progression to cancer from chronic thyroiditis.^21^ The presence of RET/PTC must not be synonymous with cancer, but rather a molecular mechanisms of carcinogenesis.^22^

Another described mechanism is the expression of p63, a tumor-suppression protein homologous to the p53, that is found in about 81% of the HT and PTC, and it is not found in normal thyroid tissues, Graves-Basedow disease or other thyroid tumors.^23^

It is questionable whether or not underlying HT is a worse prognostic factor for PTMC. Some authors suggest a less aggressive course of the disease when there is association between HT and PTC, with tendency toward a smaller tumor size, less lymph node involvement and longer survival.^15^, ^16^, ^24^ Ahn et al.^18^ reported that patients with PTC were four times more likely to have a coexisting HT compared to patients with other thyroid diseases, suggesting a link between chronic inflammation and cancer development in the thyroid gland. There was also a trend in patients with PTC and HT for a better prognosis, including smaller tumor sizes, a lower frequency of lymph node metastasis, higher disease-free and overall survival rates, than in patients with PTC alone. In a large retrospective study,^19^ 0.7% cancer-specific mortality and a 95% relapse-free 10 year survival rate were reported in patients with chronic thyroiditis compared to 5% mortality and an 85% relapse-free 10 year survival rate without chronic thyroiditis. In our study, presence of HT was detected more often particularly in patients with TMC. Microcarcinomas are occult carcinoma that are small, ≤10 mm in diameter, usually papillary in type and exhibit benign behavior. TMCs rarely metastasize to distant sites and have an excellent prognosis with a reported mortality less than 0.5%.^2^, ^25^ Furthermore, tumor size was not affected by the presence of HT. Bilateral occurrence of malignancy and HT correlated only in TMC group. Based on these results, the presence of Hashimoto's thyroiditis is not a worse prognostic factor. However, data in the literature on the relationship between PTC and HT were not primarily focused on tumor size or incidentalomas.

Papillary thyroid carcinoma (PTC) often presents as multifocal or bilateral tumors. In our study, the incidence of multifocal tumors was 12% cases in TC and 29% in TMC group. Bilateral occurrence was also higher in TMC group (20%) compare to TC group (10%). Bilateral occurrence of malignancy and HT correlated only in TMC group. On the other hand, Asanuma et al.^26^ showed that cases of concurrent diseases are more frequently multicentric (93%) when compared to cases without the association (50%).

Fine needle aspiration cytology is the most important step in the management of thyroid nodules. FNAC has a sensitivity ranging from 65% to 98% and specificity ranging from 72% to 100%.^12^ In our study, FNAC was performed in 55% of patients with nodular goiter. High sensitivity and specifity of FNAC in the diagnosis of malignant thyroid nodule was observed in both groups of patients. Interestingly, the presence of HT negatively affected the accuracy of FNAC in the diagnosis of malignancy.

## Conclusion

Hashimoto's thyroiditis is associated with an increased risk of developing thyroid carcinoma, especially thyroid microcarcinoma.

According to the high incidence of thyroid malignancy in patients with Hashimoto's thyroiditis and frequent multicentricity of these tumors, careful follow-up, clinically as well as with cytopathological study of the nodules are necessary.

## Conflicts of interest

The authors declare no conflicts of interest.
